# Esophageal reconstruction as the first step for treating secondary aortoesophageal fistula due to thoracic endovascular aortic stent infection

**DOI:** 10.1186/s44215-023-00059-w

**Published:** 2023-07-03

**Authors:** Naoto Fukunaga, Akio Shimoji, Toshi Maeda, Otohime Mori, Kosuke Yoshizawa, Kenji Minatoya, Nobushige Tamura

**Affiliations:** 1 Department of Cardiovascular Surgery, Hyogo Prefectural Amagasaki General Medical Center, 2-17-77, Higashinaniwa-Cho, Amagasaki, Hyogo 660-8550 Japan; 2 Department of Cardiovascular Surgery, Kyoto University Hospital, Kyoto, Japan

**Keywords:** Esophageal reconstruction, Aortoesophageal fistula, Thoracic endovascular aortic stent

## Abstract

**Background:**

The surgical strategy for aortoesophageal fistula (AEF) depends on the experience of each surgeon, and there is no consensus on the strategy to be adopted. We propose our two-stage operation compromising esophagectomy and reconstruction as the first step and in situ aortic graft replacement as the second step after 7 days for treating AEF secondary to thoracic aortic stent graft infection.

**Case presentation:**

A diagnosis of AEF was made in a 70-year-old man with a history of multiple aortic interventions. The patient underwent esophageal resection and reconstruction with a pedicled stomach roll endoscopically in the right thoracic cavity. Postoperatively, enteral feeding was resumed via a feeding tube placed in the jejunum to maintain adequate nutritional status. There was no evidence of either anastomotic leakage or necrosis. Seven days later, the patient underwent removal of the infected stent graft and in situ graft replacement via a redo left thoracotomy. After the surgery, the patient was able to start oral intake relatively early. Although more than 6 months has passed since the patient was discharged, no recurrence of infection has been observed.

**Conclusions:**

The benefit of our strategy is the radical treatment for secondary AEF and the early resumption of oral intake.

## Background

The strategies for aortoesophageal fistula (AEF) are mainly based on experience, and there is no universal consensus regarding the surgical strategy. We proposed a two-stage operation consisting of esophagectomy and reconstruction as the first step and in situ aortic graft replacement as the second step after 7 days for AEF secondary to thoracic aortic stent graft infection.

## Case presentation

Consent for publication was obtained from the patient, and the need for institutional review board approval was waived as it was a case report.

**Fig. 1:**
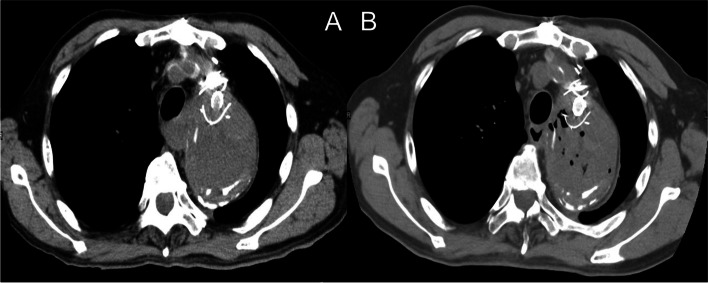
**A** Computed tomography demonstrates the possibility of stent graft infection complicated by abscess formation around the aortic aneurysmal sac. The aortic arch aneurysmal sac expanded as compared with that 8 months ago. There was no evidence of endoleak. **B** Plain CT 5 months after the initial admission shows newly developed air inside the aneurysmal sac

A 70-year-old man presented to our cardiology outpatient clinic with general fatigue lasting for a few days. His medical history included thoracic endovascular aortic repair (TEVAR) with a one-branched Inoue stent graft for acute type B aortic dissection in the 1990s, replacement of thoracoabdominal aorta with the reconstruction of visceral branches for Crawford type II dissecting thoracoabdominal aortic aneurysm in 2010, replacement of the ascending aorta and partial aortic arch for thoracic aortic aneurysm in 2020, hypertension, and hyperlipidemia. C-reactive protein (CRP) was elevated at > 10 mg/dL, and the procalcitonin level was 14.5 ng/mL. Blood cultures were negative at this point. The patient was admitted, and intravenous antibiotics were administered. Computed tomography (CT) was performed on the next day, which revealed the possibility of stent graft infection complicated by abscess formation around the aortic aneurysmal sac. The aortic arch aneurysmal sac had expanded compared with that 8 months ago, increasing from 6.6 to 7.1 cm (Fig. 1A). After 6 weeks of antibiotic treatment, the patient’s CRP level was 0.76 mg/dL, and he was discharged in a good condition.

**Fig. 2:**
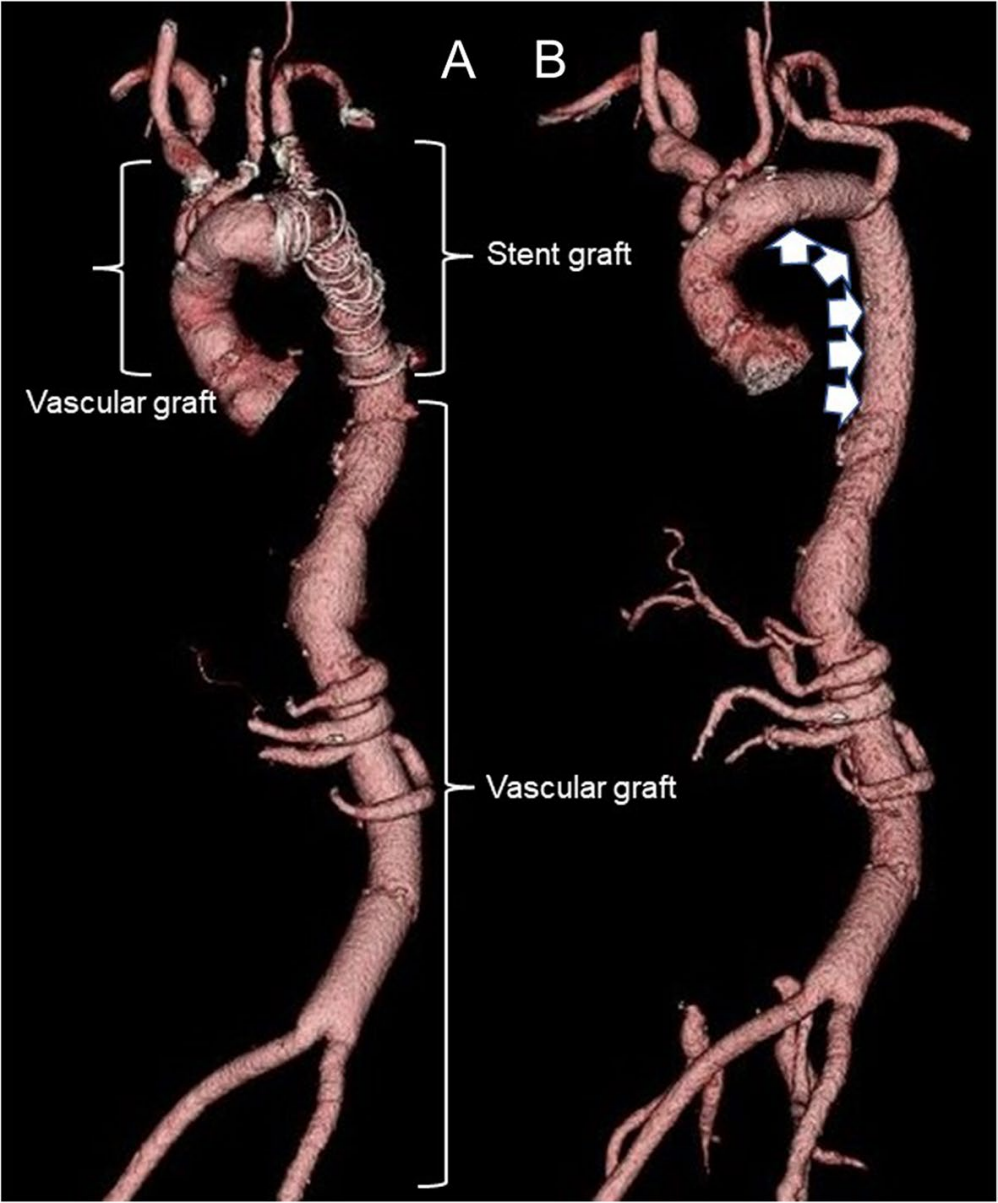
**A** Three-dimensional contrast-enhanced computed tomography shows the physical relationship between the previous grafts and the endovascular stent graft preoperatively. **B** Postoperative aortic configuration is demonstrated on three-dimensional contrast-enhanced computed tomography. Arrowheads indicate a newly implanted vascular graft. The left subclavian artery was reconstructed with a side branch after the removal of the previous stent graft

**Fig. 3:**
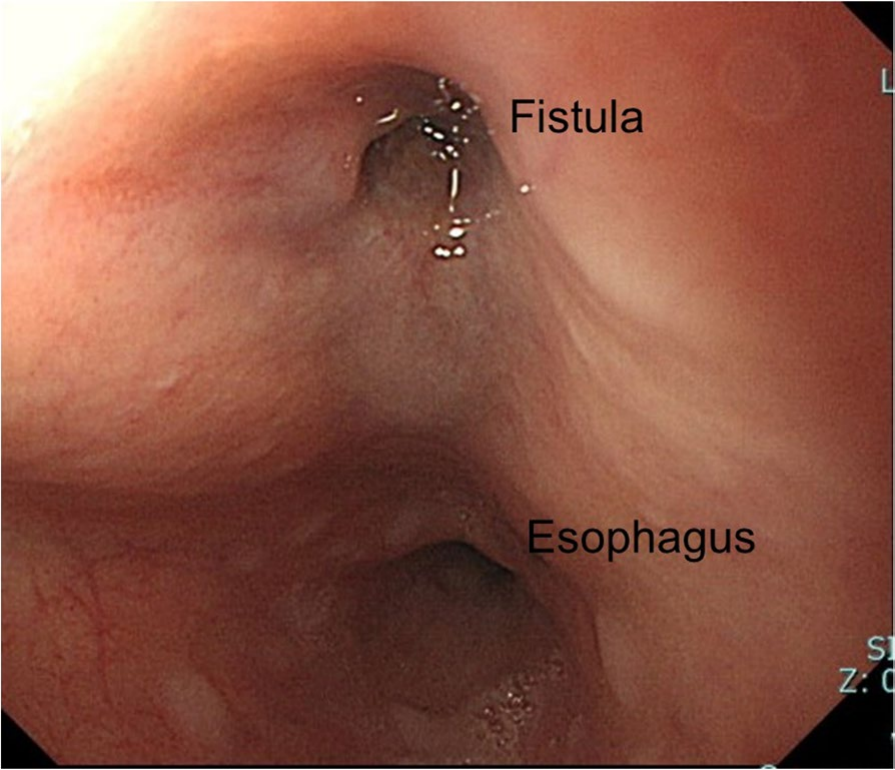
Gastroscopy revealed a 2-mm fistula in the left lateral wall of the esophagus 25 cm from the incisor

**Fig. 4:**
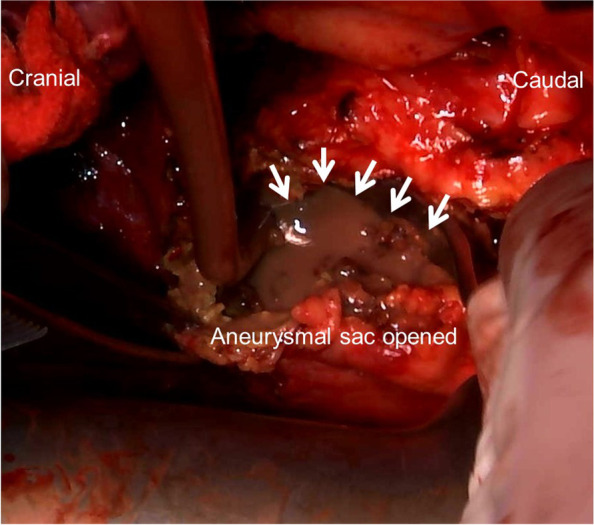
Arrows indicate a large amount of whitish pus emanating from the opened aneurysmal sac

However, 5 months later, the patient reported to the cardiology outpatient clinic with a fever lasting for 2 days. Leukocytes were slightly elevated at 8900/L, and the CRP level was 16.11 mg/dL. Plain CT demonstrated newly developed air inside the aneurysmal sac (Fig. 1B), which suggested the recurrence of infection. Three-dimensional contrast-enhanced CT demonstrated the physical relationship between the previous vascular grafts and the endovascular stent (Fig. 2A). Gastroscopy revealed a 2-mm fistula in the left lateral wall of the esophagus 25 cm from the incisor (Fig. 3). A diagnosis of AEF secondary to stent graft infection was made. As the first step in the two-stage operation, the patient underwent esophageal resection and reconstruction with a pedicled stomach roll endoscopically in the right thoracic cavity. Postoperatively, enteral feeding was resumed via a feeding tube placed in the jejunum to maintain adequate nutritional status. There was no evidence of worsening fever or wound abscess. His laboratory parameters were improved. In addition, fluid status from the chest tube appeared serous bloody and did not contain gastric contents. These findings were monitored every day by general surgeons. Overall, we confirmed that there was no evidence of either anastomotic leakage or necrosis. Seven days later, the patient underwent removal of the infected stent graft and in situ rifampicin-soaked vascular graft implantation with the reconstruction of the left subclavian artery via a redo left thoracotomy. When opening the aortic aneurysmal sac, a large amount of whitish pus emanated (Fig. 4). We were unable to perform pedicle omental wrapping due to the stomach roll in the right-sided cavity and adherence to the surrounding tissue. Intravenous antibiotics were resumed. Postoperatively, the left-sided tracheal fistula was confirmed on bronchoscopy, for which the latissimus dorsi muscle flap was successfully installed. At that time, the latissimus dorsi muscle was routed anteriorly to cover the implanted graft. Although the tracheal fistula took a while to heal and tracheostomy was required, the patient was able to start oral intake on postoperative day 58. He was transferred in a good condition to a rehabilitation facility 78 days after the redo left thoracotomy. Postoperative aortic configuration was demonstrated on three-dimensional contrast-enhanced CT (Fig. 2B). No recurrence of infection has been observed for 6 months after the redo thoracotomy.

## Discussion and conclusions

TEVAR has become an attractive alternative to open surgery for high-risk or frail patients. Some cases of AEF occur as a complication of TEVAR. Studies have shown that the incidence rate of AEF after TEVAR is 1.7–1.9%, and AEF develops 1 to 16 months after TEVAR [1, 2].

As the conservative management of AEF is associated with a high mortality rate, surgical treatment should be considered in patients who are operable. Experienced centers have proposed a single-stage surgery compromising esophageal resection, aggressive debridement, in situ reconstruction of the aorta, and omental pedicle grafting via a left thoracotomy [3–5]. Esophagectomy, graft replacement, and omental coverage are important for improving early and late survival [6].

Staged esophageal reconstruction was proposed at a mean duration of 5.5 months after esophageal resection [3, 4, 7]. During the waiting period, physicians were able to control infection, preserve swallowing function, and maintain nutritional status in affected patients. Not all survivors proceeded to the esophageal reconstruction stage after several months [3, 4].

We performed esophagectomy and its reconstruction using the stomach roll endoscopically 7 days before the removal of the infected stent graft and re-replacement of the descending aortic graft. We performed this procedure to stabilize the esophageal anastomosis, considering the fact that the redo thoracotomy approach was time-consuming to dissect severe adhesion. During the 7-day interval, we observed neither anastomotic leakage nor necrosis of the stomach roll postoperatively. Although definitions of these complications proposed by the Esophagectomy Complications Consensus Group have been widely adopted [8], a large variability of diagnostic and treatment options are available. Thus, a consensus on the diagnostic process is still lacking. For signs or presentations, fever, leukocytosis, elevated CRP, and wound inflammation are one of the indicators to suspect these complications [9, 10]. CT scan is preferred as the first-level investigation, while endoscopy is usually performed to confirm the uncertain CT diagnosis and initiate treatment [10]. In our case, the laboratory markers and clinical signs improved after the first surgery, and we decided not to have the patient examined by imaging modalities.

A CRP value around 17 mg/dL on postoperative day 3 and drain amylase level between 125 and 250 Ul/L on postoperative day 4 have been cutoff values for leakage development [9]. In addition, anastomotic leakage was detected by CT with or without endoscopy between postoperative days 2 and 7 in 76.9% of patients who developed anastomotic leakage following intrathoracic anastomosis for esophageal cancer [10]. These indicate that the 7-day interval between the first and the second surgery would be reasonable to deny the possibility of complications and proceed with the second surgery safely.

Furthermore, the patient was able to resume oral intake on postoperative day 58. Oral intake was delayed because of the treatment of the tracheal injury. The patient would have been able to resume oral intake early on after redo thoracotomy if not for the extra event of tracheal fistula. We believe that our two-stage strategy compromising esophageal resection and reconstruction endoscopically before removal of the infected stent graft was ideal for secondary AEF.

A drawback in this report is that we were unable to fully cover the prosthetic graft with the omentum during the second-stage surgery owing to the mild adhesion of the omentum around the pedicled stomach roll. Based on our experience, we recommend shortening the interval between the esophageal intervention and the second-stage surgery, for example, to 2–3 days, to fully mobilize the omentum and cover the prosthetic graft. Instead, a part of the graft was covered by the latissimus dorsi muscle at the time of installing it into the tracheal fistula [11]. In such an interval, we must deny the anastomotic leakage as there is a high possibility of the leakage before postoperative day 7 [9, 10].

A two-staged repair would be desirable because of the invasiveness of surgical procedures. Especially, in our case of redo thoracotomy, it took a few hours to dissect the left thoracic cavity and reach the aorta. In the case of hemodynamically unstable patients with arterial bleeding from the aorta through the esophageal fistula, our plan cannot be applied as bleeding control would be the priority to save a life. Of 18 patients with AEF, there were 6 patients with hemorrhagic shock, and 3 required a bridge TEVAR for the bleeding and had subsequent open surgery [4]. Although a comparative study was not possible, an experienced center advocated an aggressive approach with the late reconstruction of esophageal for only survivors who were able to tolerate the surgery [4].

## Data Availability

Data sharing is not applicable to this article as no datasets were generated or analyzed during the current study.

## Abbreviations

AEF: Aortoesophageal fistula
TEVAR: Thoracic endovascular aortic repair
CT: Computed tomography
CRP: C-reactive protein
